# Virus removal from drinking water using modified activated carbon fibers†

**DOI:** 10.1039/d1ra06373a

**Published:** 2021-09-23

**Authors:** Kamila Domagała, Jon Bell, Nur Sena Yüzbasi, Brian Sinnet, Dariusz Kata, Thomas Graule

**Affiliations:** Laboratory for High Performance Ceramics, Empa, Swiss Federal Laboratories for Materials Science and Technology Überlandstrasse 129, Dübendorf Switzerland kamila.domagala@empa.ch; Faculty of Materials Science and Ceramics, AGH, University of Science and Technology, Krakow, Poland Faculty of Materials Science and Ceramics al. Adama Mickiewicza 30 Krakow Poland; Department of Process Engineering, Eawag, Swiss Federal Institute of Aquatic Science and Technology Überlandstrasse 133, Dübendorf Switzerland

## Abstract

Activated carbon (AC) exhibits superior sorption properties compared to other porous materials, due to well-developed porous structures and high surface areas. Therefore, it is widely applied in its various forms in water purification to remove a diverse range of contaminating species. The presence of viruses in fresh water bodies poses a serious issue for human health. However, AC has not yet been commonly applied to waterborne virus removal. In this study, we present oxidation and copper impregnation treatment procedures of activated carbon fibers (ACFs) that resulted in porous structure and surface chemistry modifications. The effect of these modifications on virus removal was investigated by experimental flow studies and revealed up to 2.8 log_10_ reduction value (LRV) and 3.6 LRV of MS2 bacterio-phages for non-modified and oxidized ACFs, respectively, emphasizing the advantages of ACF surface functionalization. Copper modified fibers demonstrated a high sensitivity to media composition, resulting in a release of metal and therefore limited virucidal capacity.

## Abstract 2

Activated carbon fibers revealed better performance in filtration studies towards MS2 bacteriophage removal in comparison to activated carbon powder or granules.

## Graphical abstract

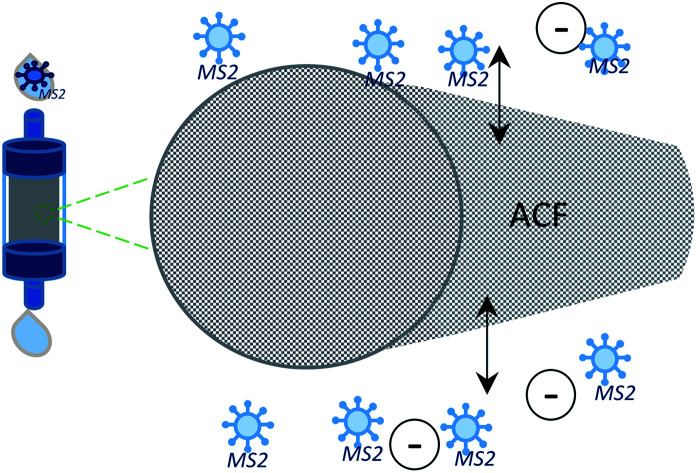

## Introduction

Water is of fundamental importance for the survival of aquatic and terrestrial organisms. Yet, water contamination keeps increasing at an alarming rate, with pathogenic viruses becoming of utmost significance for the World Health Organization (WHO) and are classified as a moderate to high threat to human health.^1^ This includes enteric viruses, which spread through drinking water and lead to serious diseases and even deaths.^1,2^ This problem especially concerns developing countries, where there is a lack of access to clean drinking water, in combination with poor sanitation and low hygiene levels, accelerating the spreading of waterborne diseases.^2^

The limitations of water treatment technologies associated with low- and middle income countries can be circumvented by the implementation of centralized treatment plants.^3,4^ Another promising option is the implementation of point-of-use (POU) systems, which allows water treatment at the consumption point.^1^ The main disadvantages of such technologies are: regular maintenance, spare parts availability, recontamination risk due to unhygienic handling after treatment and high operational costs.^2,4,5^ POU systems comprise among others, filtration-based technologies, which removes contamination not only by size exclusion, but also *via* adsorption.

Adsorbent materials for water technologies should exhibit good chemical and mechanical-stability, a large surface area and a developed porous structure, modified surface chemistry for enhanced adsorbate–adsorbent interactions, fast adsorption kinetics and the potential for reuse-regeneration.^6^ Adsorbent materials for water treatment might display a naturally developed porous structure or they may require physical or chemical activation in order to optimize the porous structure for a particular adsorption application. Natural adsorbents are those such a s clay minerals,^7^ natural zeolites,^8^ oxides^9^ and biopolymers.^10,11^ The engineered adsorbents include various carbon derived materials (*i.e.* activated carbon (AC), carbon fibers, carbon nanotubes and graphene)^12,13^ and inorganic based materials (such as zeolites (silica or alumina based), layered double hydroxides^14^ or metal organic frameworks^15^).^6,11^ There are typically two modes of operation for adsorption based separation, namely batch adsorption and solid–liquid separation, which includes decantation, filtration and centrifugation.^6^

Activated carbon is one of the most widely applied adsorbent materials, which is utilized in plethora of different technologies (food processing, industrial waste gas separation, volatile organic compounds removal),^16^ and also in waste-water and drinking water treatment.^17,18^ AC in water purification is mainly used in its unmodified or oxidized form for various pollutant removal application, *i.e.* removal of taste/odor compounds, pesticides, dyes, organic contaminations and heavy metals.^11,16,19^ AC can also be modified with metal species/metal oxides in order to increase the amount adsorbed of the target species from both the gas and liquid phase.^20–23^

AC is not commonly used for virus removal, exhibiting rather low affinity towards them.^23–26^ Conversely, Matsushita *et al.* reported up to 4 log_10_ reduction value (LRV) in long-term batch studies using grained powdered activated carbon (PAC).^27^

Activated carbon fibers (ACFs) are characterized by a large adsorption capacity due to a high surface area and a well-defined porous structure (wide pore size distribution), which are important characteristics for water treatment.^17,18,28^ ACFs exhibit 2–50 times higher adsorption rates compared to other AC forms, such as powder or granular (GAC) activated carbon, due to a smaller diameter, higher microporosity and larger surface area.^29^ ACFs can be functionalized using several different approaches (*e.g.* plasma, chemical oxidation) in order to optimize the porous structure and surface chemistry, resulting in an improved sorption capacity for target species.^30,31^ The ACFs surface can be modified, when functionalization is performed, and characteristics such as roughness and porosity can increase, which effectively leads to an increase in adsorption active sites.^30,32^ Additionally, incorporated oxygen functional groups increase chemical reactivity and hydrophilicity.^30^ The advantages of ACFs include low hydrodynamic resistance,^16^ thermal stability beneficial for material thermal regeneration, as well as a prolonged lifetime, and high Hamaker constant,^33^ which are important properties for virus particles adsorption and retention.

This study focuses on the development of activated carbon fibers that have undergone surface modification by oxidation treatment, followed by Cu-impregnation under controlled pH conditions. The role of porous structure and surface chemistry on the removal of viruses from contaminated drinking water at the point of consumption have been investigated. The postulate that virucidal copper species^34,35^ create additional adsorption sites for microbes has been investigated. The applicability of carbon fibers and their composites in virus removal was tested with MS2 bacteriophages, an enteric virus surrogate.^36^

## Experimental

### Material preparation

Commercial activated carbon fibers (ACF-1603-20) were purchased from Kynol Europa GmbH, high purity 65% HNO_3_ from Carl Roth, and Cu(NO_3_)_2_·3H_2_O from Merck. All solutions were prepared in ultrapure water (MicroPure UV System), Thermo Scientific (resistivity 18.2 MΩ cm). Gases were obtained from PanGas: for porous structure characterization CO_2_ (99.995%), N_2_ (99.995%) were used; for thermal treatment 2 vol% H_2_ in Ar and N_2_ ≥99.995% were used; for samples degassing N_2_ ≥99.995% was used. Chemicals used for Boehm titrations included 0.05 M HCl, 0.05 M NaOH, Phenolphthalein from Carl Roth, Na_2_CO_3_ (p.a. ≥99.7%), NaHCO_3_ (p.a. ≥99.5%) from Merck. Chemicals used for point of zero charge measurements included: NaCl (p.a. ≥99.0%) from Merck. The chemicals used for the Double Agar Layer method were: CaCl_2_ (Ph. Eur.) from Merck, C_6_H_12_O_6_, and NH_2_C(CH_2_OH)_3_ from VWR Chemicals, MgSO_4_·7H_2_O (p.a. ≥98.0%) from Fluka, Tryptic Soy Agar from Difco, Tryptone and Yeast Extract from Carl Roth.

#### Functionalization of ACFs

The copper-impregnated activated carbon fibers evaluated in this work were obtained by a tailored combination of HNO_3_ oxidation and Soxhlet extraction, followed by copper impregnation by liquid-phase adsorption. Briefly, as-received activated carbon fibers (ACF_AR_) (0.015 g mL^−1^) were immersed in 65% HNO_3_ and heated for 60 minutes at 90 °C under reflux. The resulting oxidized ACFs (ACF_OX_) were rinsed with water to neutral pH, and oven-dried overnight at 60 °C. A portion the ACF_OX_ were extracted using a Soxhlet apparatus (ACF_OX+SOX_) to remove any residual acidic. ACF_OX_ was placed in an extraction thimble (Cellulose thimbles 603, Whatman) and water-refluxed for 72 h, until a constant pH was achieved and named ACF_OX+SOX_. The ACF_OX+SOX_ were then dried in oven at 60 °C for 48 h. Additionally, a reference sample was produced and consisted of as-received fibers purified with Soxhlet, designated with the code ACF_AR+SOX_ and were used to study the effects of Soxhlet extraction on the porous structure and surface chemistry.

#### Copper-impregnated ACFs

All functionalized samples were pre-screened to determine the implications of different treatment procedures on porous structure and surface chemistry modifications, which are crucial factors in liquid-phase adsorption. Based on the samples characterization, ACF_OX+SOX_ was selected for copper-impregnation studies. The non-modified ACF_AR_ was also subjected to copper-impregnation, and used as control specimen to understand the advantages of oxidation-Soxhlet pretreatments.

The copper-impregnation was achieved by mixing (200 rpm, stir plate), at room temperature for 24 h, 50 mg of either ACF_AR_ or ACF_OX+SOX_ with 25 mL of different initial concentrations (*C*_init_: 0.05, 0.1 0.5, 1, 2, 2.5, 5, 10, 20 mmol) of Cu(NO_3_)_2_·3H_2_O solution. ACFs were vacuum filtered (PVDF membrane 0.1 μm, 47 mm, Hawach Scientific Co., Ltd) and the concentration of permeated copper was determined by ICP-MS (ICP-MS 7500CE, Agilent). A series of initial pH conditions (pH_init_: unmodified– 4.3, 4.0, 2.0 (only for ACR_OX+SOX_), 1.0) were tested during the adsorption process. The resulting optimal combination of Cu^2+^ concentration of 20 mmol and pH_init_ equal to 4.0 were used for the preparation of two composites: (i) Cu-impregnated sample oven-dried at 60 °C (CuACF_OX+SOX_); (ii) H_2_ treated sample (HCuACF_OX+SOX_) at 350 °C under H_2_/Ar atmosphere for 2 h with heating rate 5 °C min^−1^ in a tube furnace (GHC 120900, Carbolite Gero GmbH & Co. KG).

### Material characterization

#### Porous structure and surface area

Porous structure and surface area were determined using a Micromeritics ASAP 2020 volumetric adsorption apparatus by adsorption of N_2_ at 77 K and CO_2_ at 273 K.

#### Elemental analysis (EA)

Elemental analysis (EA) was performed on a CHNS/O Flash Smart, Thermo Scientific instrument.

### Surface group characterization

#### Point of zero charge

Point of zero charge (pH_pzc_) was measured according to the procedure described by Stoeckli *et al.* called pH drift method.^37^

#### Boehm titration

Boehm titrations were used to identify and quantify the amount of incorporated oxygen functional groups on the carbon surface.^38,39^

#### X-ray photoelectron spectroscopy (XPS)

X-ray photoelectron spectroscopy (XPS) were acquired using hemispherical analyzer EA15 (PREVAC) equipped with a dual anode X-ray source RS40B1 (PREVAC).

### Carbonaceous structure and morphology

#### Raman spectroscopy

Raman spectroscopy was performed with Renishaw Raman System H45383 with Spectra-Physics laser (Ar *λ* = 514 nm, 10% laser power of 24 mW, 30 second exposure) to determine the carbonaceous molecular structure.

#### Scanning electron microscopy (SEM)

A FEI Nano-SEM 230 system was used to determine the surface morphology of the carbon substrates and their composites.

#### X-ray diffraction (XRD)

X-ray diffraction (XRD) was used to determine the phases of the obtained composites using a PANalytical X'Pert PROh-2h, Malvern scan system equipped with a Johansson monochromator (CuKa1 radiation, 1.5406 Å) and a X'Celerator linear detector.

More detailed descriptions of sample preparation and measurement details of particular characterization methods are summarized in the ESI.†

### Conditioning and MS2 removal tests of ACFs and composites

#### Conditioning test

300 mg of composite or ACFs was placed in a specially designed cartridge (details given in ESI†), and cut glass fiber filters (0.4 μm, Macherey–Nagel) were placed in the inlet and outer caps of cartridge to avoid the release of fibers from the system. The prepared cartridge was rinsed with 0.01 M NaCl solution with the pH adjusted to 5.0 and 7.0 for 24 h at a flow rate of 150 mL h^−1^. Permeate samples were regularly collected and the concentration of copper in permeate was analyzed *via* ICP-MS (ICP-MS 7500 CE, Agilent). All tests were performed in duplicate.

#### MS2 bacteriophage removal experiment

The ready to use MS2 stock solution and its *Escherichia coli* host were purchased from the Culture Collection of Switzerland. The virus removal experiments were performed in a laboratory-scaled experimental setup by passing 200 mL of 10^5^ PFU mL^−1^ MS2 bacteriophages solution through conditioned cartridges using a peristaltic pump. The permeates were collected and analyzed for (i) MS2 concentration using Double Agar Layer method^40^ (the composition of solutions used are summarized in Table S1†) and (ii) the presence of potentially dissolved copper using ICP-MS. The analysis of MS2 concentration was done directly after permeate collection (*t* = 0 h), and after 2 hours of storage (*t* = 2 h) to assess additional inactivation over time. The MS2 removal test was performed at a pH of 5.5 and 7.3, and also in duplicate with negative and positive controls alongside each experiment. MS2 log_10_ removal (LRV) was calculated using the following formula:1
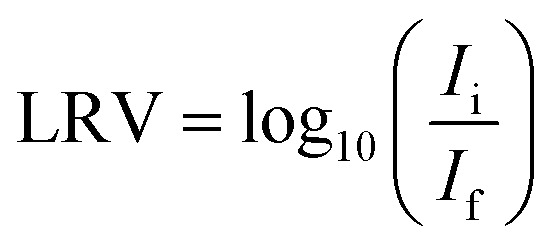
where, *I*_i_-the initial MS2 concentration (PFU mL^−1^) and *I*_f_-the MS2 concentration in permeate (PFU mL^−1^).

## Results and discussion

### Characterization of functionalized ACFs

**Fig. 1:**
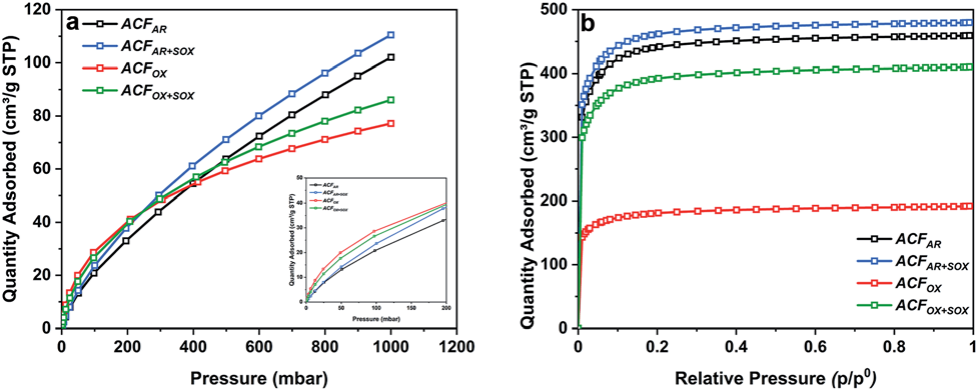
(a) CO_2_ adsorption isotherms at 273 K and (b) N_2_ adsorption isotherms at 77 K of ACFs.

Table S2† summarizes the specific surface areas and porous structure of ACFs assessed by CO_2_ adsorption at 273 K (Fig. 1a) and N_2_ at 77 K (Fig. 1b). The Dubinin–Radushkevich equation^41^ was applied to calculate the micropore volumes (V_CO_2__ <0.7 nm), revealing a high microporosity of the fibers. ACF_AR_ and ACF_AR+SOX_ exhibit a micropore volume equal to 0.108 cm^3^ g^−1^, and 0.130 cm^3^ g^−1^, respectively, while oxidized samples demonstrate 25% microporosity increase (0.137 cm^3^ g^−1^, 0.141 cm^3^ g^−1^ for ACF_OX_ and ACF_OX+SOX_, respectively).

According to the IUPAC classification, the N_2_ adsorption isotherms are type I,^42^ and the Langmuir equation^43^ was used to calculate the maximum amount adsorbed and the total pore volume (V_N_2__) of the carbon materials, obtaining 0.453 cm^3^ g^−1^ and 0.468 cm^3^ g^−1^ for ACF_AR_ and ACF_AR+SOX_, respectively. Oxidation reduces V_N_2__ to 0.186 cm^3^ g^−1^ (ACF_OX_), while Soxhlet purification causes as light reduction in the N_2_ total pore volume to V_N_2__ = 0.397 cm^3^ g^−1^, compared to the ACF_AR_ sample. Results prove that Soxhlet extraction is opening up the porosity and at the same time does not greatly change the porous structure between purified samples. The micropore radius, calculated based on N_2_ adsorption, remains almost unchanged and is independent of the treatment, varying around 1 nm. Values obtained for CO_2_ adsorption revealed an increase of micropore mean radius from 0.26 nm to 0.79 nm for ACF_AR_ and ACF_OX_, respectively. The details about pore size distribution are enclosed in ESI.†

The specific surface area of ACF_AR_ is equal to 1677 m^2^ g^−1^, and this decreased by 60% to 698 m^2^ g^−1^ for ACF_OX_. Such a decrease might be a result of micropore blocking due to newly introduced surface oxygen complexes or formation of humic substances.^44,45^ Further explanations suggest surface smoothing^46,47^ or collapse of the pore walls due to the strong treatment conditions used.^48^ Soxhlet purification significantly increases SSA up to 1652 m^2^ g^−1^ for ACF_OX+SOX_. Elemental analysis results collected in Table 1 reveal significant changes in elemental content. Oxidation led to nearly a 40% carbon decrease and approximately 4 times increase in oxygen content. The nitrogen content increased from 0 (the value below detection limit) to 0.59 wt%, which did not remarkably decrease with the Soxhlet purification. This suggests that nitrogen species are strongly bonded to ACFs and were not removed.

**Table:** Elemental analysis and atomic% of elements obtained from X-ray photoelectron spectroscopy survey spectra of ACFs

Carbon sample	EA (wt%)				XPS survey (at%)		
	C	H	N	O	C	O	N
ACF_AR_	90.6	0.5	0.0	8.1	92.6	6.4	1.0
ACF_AR+SOX_	91.2	0.7	0.0	5.6	91.2	8.1	0.7
ACF_OX_	64.8	1.1	0.6	32.9	79.5	18.3	2.2
ACF_OX+SOX_	65.0	1.0	0.4	29.7	80.6	17.6	1.8

Analysis of XPS Survey spectra (Table 1) reveals a three fold increase in oxygen and two fold increase in nitrogen content after oxidation. Fig. S1† presents the XPS spectra of all ACFs types, while Table 2 shows the analysis of surface functional groups.

Carbon, oxygen and nitrogen functional group analysis from X-ray photoelectron spectroscopy

**Table:** 

Carbon sample	Components from C 1s profile (at%)				
	C sp^2^	C–O + C–N	C=O	COOH	CO_3_
ACF_AR_	71.4	8.4	4.4	4.8	3.6
ACF_AR+SOX_	68.0	10.7	4.6	4.9	3.0
ACF_OX_	59.4	5.9	3.3	8.8	2.2
ACF_OX+SOX_	58.9	6.5	3.3	9.8	2.1

**Table:** 

Carbon sample	Components from O 1s profile (at%)		
	OH/C=O	COOH/O–C + H_2_O	O–C=
ACF_AR_	1.4	4.0	1.0
ACF_AR+SOX_	1.0	6.6	0.5
ACF_OX_	6.5	10.7	1.1
ACF_OX+SOX_	6.9	9.1	1.6

**Table:** 

Carbon sample	Components from N 1s profile (at%)				
	N–6 (pyridinic) or N–metal, metal–CN	N–5 (pyrrolic) or N–C	(Quaternary) N–Q/NH_4_^+^	(Pyridinic N oxides) N–X/N^3+^-O, NO_2_^−^	N^5+^–O–C, NO_3_^−^
ACF_AR_	0.2	0.5	0.2	0.1	0.0
ACF_AR+SOX_	0.0	0.5	0.1	0.0	0.1
ACF_OX_	0.1	0.3	0.4	1.2	0.2
ACF_OX+SOX_	0.2	0.4	0.4	0.8	0.1

Acid treatment resulted in an increase of carboxylic, hydroxyl and nitrogen functional groups on the ACFs surface. After both treatment steps, a decrease in nitrile groups concentration and increase in concentration of pyridinic oxides: N^3+^–O–C; NO_2_^−^ and nitrate (iii) groups was noted.^45^ Results of oxygen content detected with XPS and EA techniques follow the same trend, although XPS only probes the outermost surface, while EA gives the chemical composition of the entire bulk. This indicates that the effect of oxidation is relatively uniform; however, the nitrogen functional groups are more prevalent on the outermost surface, rather than inside fibers porous structure.

Boehm titration results summarized in Table S3† demonstrate an increase of the total amount of functional groups from 0.46 mmol g^−1^ for ACF_AR_, to 3.31 mmol g^−1^ for ACF_OX_ with a significant increase in the concentration of carboxylic groups (2.26 mmol g^−1^).

Comparison of the pH_PZC_ of fibers, presented in Fig. S2,† shows similar values for as-received fibers: 5.8 and 5.6 for ACF_AR_, and ACF_AR+SOX_, respectively. HNO_3_ treatment resulted in the pH_PZC_ shifting to 1.9 for ACF_OX_ that implies a negative surface charge in aqueous solution, due to the presence of acidic functional groups that release the protons.^45^ Soxhlet purification slightly decreases the pH_PZC_ to value of 1.5 (ACF_OX+SOX_).

Raman spectroscopy of ACFs, presented in Fig. S2,† shows the characteristic D (1344 cm^−1^) and G (1599 cm^−1^) bands of the analyzed materials: the G band is assigned to graphitic in-plane vibrations with *E*_2g_ symmetry, while the D band corresponds to the presence of graphitic the defects and disorders.^49^ Both peaks are very prominent and narrow indicative of a low defective level of the material's carbon structure.^50^ The second order frequency is split into three bands: G′ (2715 cm^−1^), D + G (2920 cm^−1^) and 2D′ (3200 cm^−1^), suggesting a highly crystalline ordered material.^50^

Raman spectra are overlapping, indicating that the applied treatment has no significant effect on material structure. Detailed peak analysis (Table 3) confirms almost no difference between the band positions, while the *I*_D_/*I*_G_ intensity ratio slightly decrease within treatment. The *I*_D_/*I*_G_ ratio not only represents the exact change in structure order but also the contribution of heteroatoms introduced during functionalization (O, H, N) that heavily influence the Raman spectra.^51^

**Table:** Peak intensity ratio, peak area ratio, half of the maximum peak width, peak position values of the first-order D and G band of activated carbon fibers

Carbon sample	*I*_D_/*I*_G_	*A*_D_/*A*_G_	WD, cm^−1^	WG, cm^−1^	D-Peak position, cm^−1^	G-peak position, cm^−1^
ACF_AR_	0.87	2.12	159.0	64.5	1344.0	1599.0
ACF_AR+SOX_	0.82	1.81	159.0	71.7	1351.7	1600.3
ACF_OX_	0.84	1.90	177.6	78.7	1352.3	1595.7
ACF_OX+SOX_	0.92	2.08	147.0	61.6	1342.0	1599.0

**Fig. 2:**
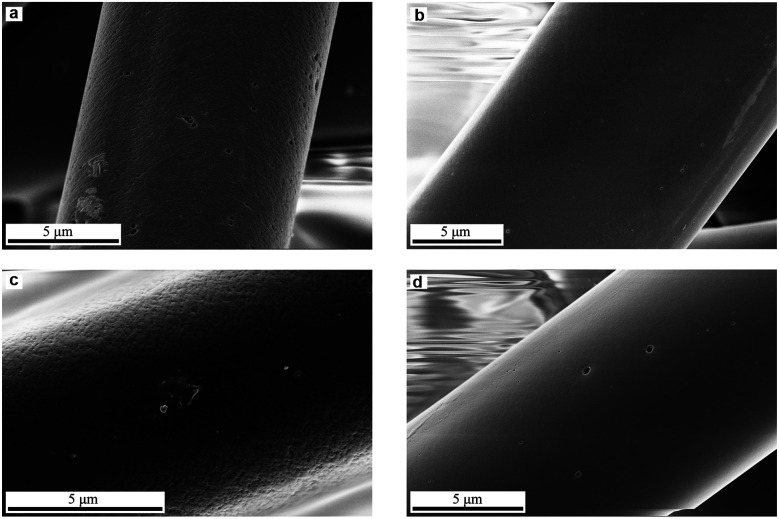
SEM images of (a and b) ACF_AR_, (c and d) ACF_OX_.

Fig. 2 shows SEM images of the ACF_AR_ and ACF_OX_. ACF_AR_ exhibit a surface with heterogeneous morphology; as some fibers have a smooth, regular surface while others possess grooves, which could be interpreted as macropores.

The fiber's diameter is in the range 9–18 μm, while length 1.5–4.5 mm. ACF_OX_ SEM images indicate no structural morphological changes, as the fibers exhibit the above-mentioned difference in morphology confirming heterogeneity of the material, even after acid treatment. No change in fibers diameter and length was observed after the oxidation treatment.

### Copper-impregnated ACFs

The adsorption of metal ions on AC from aqueous solution is dependent on a number of various factors including metal ion concentration, pH dependent speciation, solution pH, contact time, temperature, amount of carbon used and its surface functionality.^52^ The amount of metal ions adsorbed on the ACFs surface was determined using the following equation:2
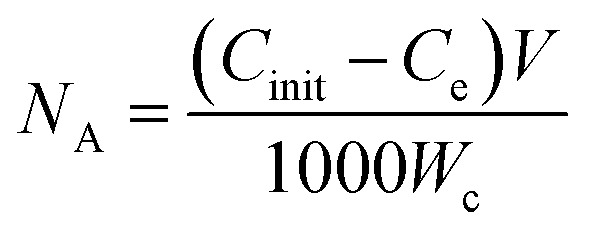
where: *N*_A_-the amount of metal ions adsorbed (mmol g^−1^), *C*_init_ and *C*_e_-the initial and equilibrium concentrations of metal ions (mmol), respectively, *V*-the volume of solution (L), *W*_c_-the weight of ACFs (g).

**Fig. 3:**
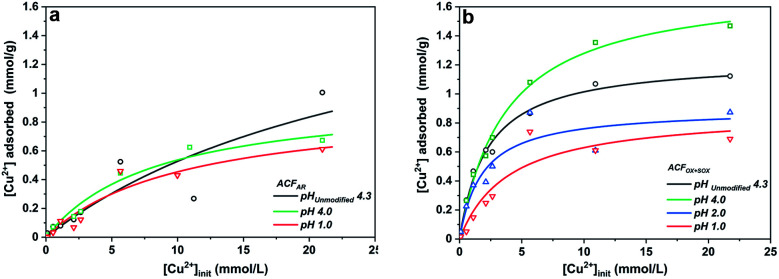
Adsorption isotherms of Cu_(aq.)_^2+^ adsorbed onto (a) ACF_AR_; (b) ACF_OX+SOX_ at 298 K.

The adsorption of Cu_(aq.)_^2+^ onto ACFs can be described by the Langmuir equation,^53^Fig. 3 presents the adsorption isotherms for ACF_AR_ and ACF_OX+SOX_, while the Table S4† shows the parameters derived from Langmuir equation fitting. The adsorption constant (*K*) is greater for fibers that possess a higher concentration of carboxylic and hydroxide functional groups, while the maximum amount of copper adsorbed (*n*_m_) exhibits a strong pH dependence, obtaining higher values when solution pH_init_ is higher than the fibers pH_PZC_.

**Fig. 4:**
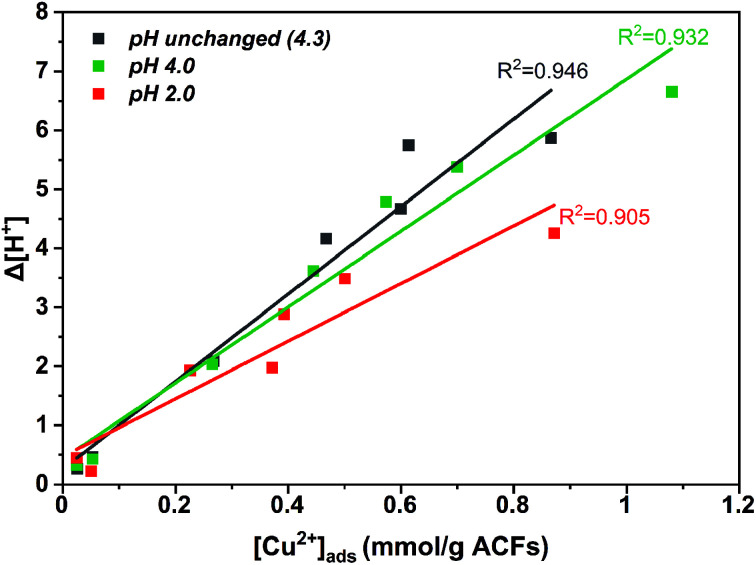
Δ[H^+^] displacement *vs.* [Cu^2+^]_(ads)_ adsorbed on functionalized + Soxhlet activated carbon fibers.

A higher copper uptake was observed for ACF_OX+SOX_ (9.3 wt%) compared ACF_AR_ (4.3 wt%), which was linked to the presence of functional groups (carboxylic and hydroxide groups) and pH_PZC_,^52^ also a higher *C*_init_ led to increase in adsorption, due to a greater driving force by a concentration gradient. Adsorption of Cu^2+^ results in final pH drop, due to the release of hydrogen ions from the surface functional groups indicating an ion exchange mechanism.^52,53^ This is demonstrated by a clear correlation between the amount of protons released and the amount of copper ions adsorbed for ACF_OX+SOX_ when pH_init_ > pH_PZC_ (Fig. 4).

This dependence is valid only up to [Cu]_init_ =5 mmol. Furthermore, it is possible that at higher pH values, copper ions are adsorbed to nitrogen functional groups by a coordination mechanism, as at low pH nitrogen groups might be protonated.^53^ The aforementioned observations indicate that the adsorption is assigned with chemical interactions described by an ion exchange mechanism, involving the displacement of protons after Cu coordination. It suggests that the following surface structures might be formed during copper adsorption:^52^

3 >C–COOH + Cu^2+^ → C–COOCu^+^ + H^+^

4 (>C–COOH)_2_ + Cu^2+^ → (>C–COO)_2_Cu + 2H^+^

5 >C–OH + Cu^2+^ → >C=ȮCu^+^ + H^+^

6 >N: + Cu^2+^H_2_O → >N–Cu(OH)^+^ + H^+^

As-received ACFs did not in display a correlation between desorbed protons and adsorbed copper ions, as they possess insufficient oxygen and nitrogen functional groups for Cu coordination.

**Fig. 5:**
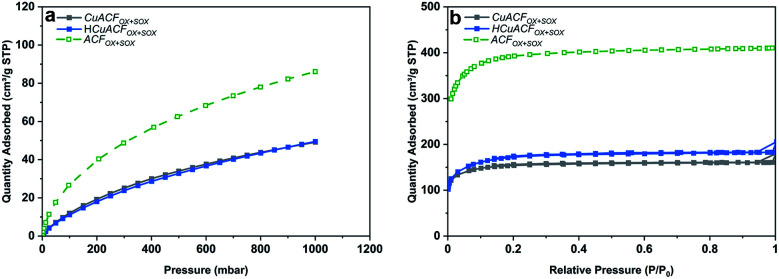
(a) CO_2_ adsorption isotherms at 273 K; (b) N_2_ adsorption isotherms at 77 K of activated carbon fibers based composites.

These results demonstrate that an enhanced adsorption of aqueous metal ions occurs on ACFs due to functionalization followed by Soxhlet extraction. Thus, the ACF_OX+SOX_ fibers and selected synthesis conditions of [Cu]_init_ = 20 mmol and pH_init_ equal to 4.0 were used for the preparation of two composites (i) CuACF_OX+SOX_ (Cu-impregnated sample oven-dried at 60 °C) and (ii) HCuACF_OX+SOX_ (Cu-impregnated sample treated at 350 °C under H_2_/Ar atmosphere). Results of the Cu-composites porous structure characterization are collected in Table S5.† The CO_2_ adsorption isotherms are shown in Fig. 5a and reveal that for the obtained composites, micropore volume and total pore volume (Fig. 5b) decreased by ∼40–55% in comparison to the ACF_OX+SOX_, due to pores blockage by adsorbed copper. In the considered pH range, mainly Cu^2+^ occurs (Fig. S4†), with an ionic radius equal to 0.70 Å,^54^ the radius of other possible species Cu(H_2_O)_6_^2+^ is 0.62–0.94 Å,^55^ thus it can easily be adsorbed in pores of ACFs. This phenomenon together with other techniques, *e.g.*, Boehm and XPS, allowed for pores and surface analysis, and confirmed copper species adsorption onto ACFs surface and in the pores.

Analysis of the surface chemistry using XPS proves the presence of copper on the ACFs surface and reveals bonding to the oxygen and nitrogen functional groups, but preferentially to the CN groups (Fig. S5 and Table S6†). Additionally, treatment under a H_2_/Ar atmosphere led to the conversion of the functional groups and changes in copper bonding, as the stability of the functional groups is thermally dependent.^56,57^

XRD analysis (Fig. S6†) indicates the presence of a Cu phase in HCuACF_OX+SOX_ composite, while the calculated^58^ crystallite size is equal to 10.5 nm. For the CuACF_OX+SOX_ sample, no copper species were detected, as the metal ions might possibly adsorb mainly in the fiber pores or are not in an amorphous phase. Additional ICP-MS analysis (ESI) of obtained composites revealed a copper content within the samples of 59.7 mg_Cu_ g^−1^ of sample 70.3 mgCu/g of sample for CuACF_OX+SOX_ and HCu ACF_OX+SOX_, respectively. These results are in accordance with the calculations obtained using eqn (2). The fibers used for synthesis (ACF_OX+SOX_) contain 0.51 mg_Cu_ kg^−1^ of sample, while ACF_OX_ contains 1.44 mg_Cu_ kg^−1^ of sample.

The pH_PZC_ of CuACF_OX+SOX_ and HCuACF_OX+SOX_ presented on Fig. S7† is equal to 3.4 and 6.9, respectively. The pH_PZC_ was shifted towards higher values due to copper species incorporation, while thermal treatment increased the pH_PZC_ value further. This can be explained due to functional groups decomposition^59^ and also the presence of copper species with different oxidation states.^60^

**Fig. 6:**
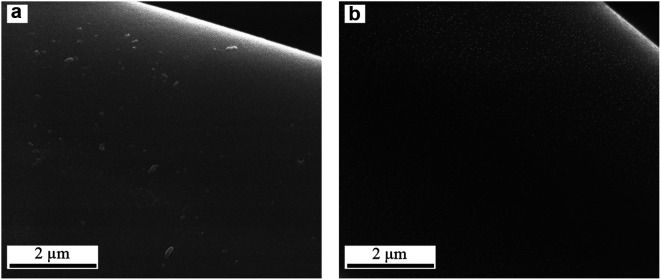
SEM images of activated carbon fibers based composites (a) CuACF_OX+SOX_; (b) HCuACF_OX+SOX_.

SEM images of composites are depicted in Fig. 6, CuACF_OX+SOX_ exhibits low amount and random distribution of copper species, while in HCuACF_OX+SOX_ sample fibers are uniformly covered by a dense layer of fine Cu particles.^61^

### Stability study of obtained composites

Carbon substrates and composites were rinsed for 24 h with 0.01 M NaCl at pH 5.0 and 7.0, while the copper concentration was monitored in regularly collected permeate samples.

**Fig. 7:**
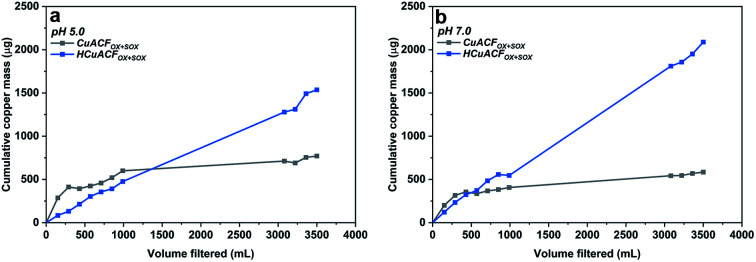
Results of conditioning of ACFs composites at (a) pH 5.0 (b) pH 7.0.

Fig. 7a and b ‡ ‡The presented data come from duplicate measurements, statistics and standard error cannot be presented. The results of both tests rounds are comparable. shows the dynamic release of copper as a function of filtered volume at pH 5.0 and 7.0. The values represent the cumulative mass of desorbed copper in the total collected permeate volume. It can be observed, that the copper amount released was higher at the beginning, then gradually decreased and saturated for CuACF_OX+SOX_, while HCuACF_OX+SOX_ exhibited not only a higher level of released copper but also a constant increase with the same ratio over time. The stability of the Cu-composites is affected by media pH, due to enhanced copper solubility under acidic pH values.^62^

CuACF_OX+SOX_ stability curves saturated after passing ∼3000 mL of solution at both pHs. At pH 5.0, the amount of copper released reached 584 μg, which represents 2 wt% of the total copper content in the sample. At pH 7.0, a 30% increase in copper release was observed in comparison to pH 5.0. The obtained value was 771 μg, which makes up 3 wt% of total copper.

The trend in HCuACF_OX+SOX_ copper release increased progressively with each water volume passed. This indicates either weak copper-fiber bonding or that the stabilization time was too short in order to reach thermodynamic equilibrium. It leads to the conclusion of further or even constant copper release, which demonstrates the destructive impact of thermal treatment onto bonding strength. At pH 5.0, the released copper amount was equal to 2088 μg (7 wt%), while at pH 7.0 it was ∼30–40% lower, reaching 1536 μg, which is equal to 4 wt% of total copper present in the sample.

The presented data indicates the enhanced performance of the CuACF_OX+SOX_ composite and highlights the instability of the HCuACF_OX+SOX_. An unstable material, such as HCuACF_OX+SOX_, might have a limited lifetime and pose deleterious health and environmental implications. The presented data indicate that heat treatment of the composite materials results in the conversion of the functional groups^59^ and re-organization of bonding types between copper and fibers.

### MS2 bacteriophage removal test of ACFs and their composites

The MS2 bacteriophages removal studies were conducted after ACFs conditioning. The MS2 concentration was quantified directly after permeate collection (*t* = 0 h) and again after a 2 hour storage (*t* = 2 h) period at room temperature. Additionally, the copper concentration in the permeate was measured, to eliminate the effect of continued MS2 inactivation due to metal presence that impacts LRV.^12,63^

### ACF_AR_ and ACF_OX+SOX_

**Fig. 8:**
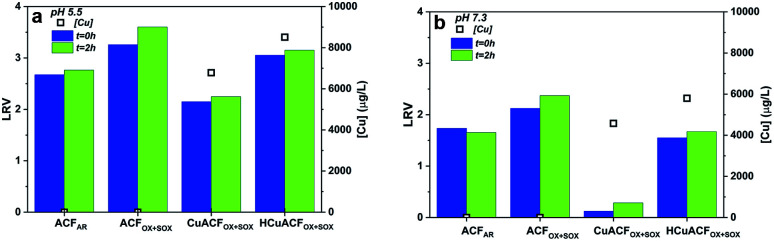
Results of MS2 removal test of ACFs and their composites at (a) pH 5.5 (b) pH 7.3.

The results of the MS2 removal study at pH 5.5 and 7.3 are shown in Fig. 8a and b‡. ACF_AR_ showed a LRV equal to 2.7 at pH 5.5, while at pH 7.3, the LRV was equal to 1.7. Values recorded at *t* = 0 h remained almost unchanged in comparison to those obtained after 2 h of storage (*t* = 2 h): 2.8 and 1.7, respectively. The copper concentration in the permeate was equal to 1.2 μg L^−1^ at pH 5.5 and 2.0 μg L^−1^ at pH 7.3, which did not result in additional MS2 inactivation over time.

The ACF_OX+SOX_ virucidal performance was 20% higher in comparison to ACF_AR_, equal to 3.3 LRV at pH 5.5 and 2.1 LRV at pH 7.3, which slightly increased after 2 h of permeate storage to 3.6 LRV at pH 5.5 and 2.4 LRV at pH 7.3, respectively. The permeate Cu concentration was very low – 0.05 and 0.03 μg L^−1^ at pH 5.5 and 7.3, respectively. The greater virucidal activity of oxidized fibers suggest that the oxidation treatment and the associated changes introduced into material (porosity opening, functional groups presence) are profitable for virus adsorption. Additionally, the higher virus removal at lower pH might be justified by the tendency of MS2 to agglomerate at acidic pH values.^64^

The virucidal properties presented by ACF_AR_ and ACF_OX+SOX_ were higher than those shown by the granular or powder form of AC. In the filtration test, Hijnen *et al.* obtained 0.2–0.7 LRV of MS2 bacteriophages for GAC at pilot scale,^24^ Shimabuku *et al.* noted 0.32 LRV for 150 g of GAC,^23^ Gerba *et al.* reported up to 50% of Poliovirus removal for GAC,^26^ while Cookson *et al.* observed <1 LRV.^25^ In the batch studies, Gerba *et al.* observed up to 92% of Poliovirus removal for GAC,^26^ Matsushita *et al.* noted up to 4 LRV for super-powdered AC, depending on the AC particle size, contact time and solution ionic strength.^27^

The contribution of following forces were suggested as possible mechanisms responsible for virus removal: electrostatic interactions, van der Waals forces, hydrophobic and steric repulsions.^65,66^ However, depending on the filtration material specific forces could have higher contribution in virus removal than the other. Adsorption by van der Waals forces are influenced by a fibers great specific surface area and high Hamaker constant.^65,67^ Well-developed porosity is also profitable, nevertheless the ACFs pores are too small to retain viruses. The higher MS2 removal by ACF_AR_ at pH 5.5 in comparison to pH 7.3 is in accordance with electrostatic adsorption.^68^ At pH 5.5 electrostatic interactions might dominate the removal process, as the pH_PZC_ of ACF_AR_ is 5.8, while for MS2 is equal to 3.9, therefore surfaces are oppositely charged. At pH 7.3, both fiber and bacteriophage surfaces are negatively charged; hence, electrostatic adsorption is assumed not to be dominant for retention. The same situation occurs at both tested pHs conditions for ACF_OX+SOX_ (pH_PZC_ 1.5), which possess negative charge. Therefore, it can be deduced that there can be other forces responsible for virus removal. The hydrophobic effects can be one of main contributors to virus retention, due to the hydrophobic character of both MS2 and ACFs, that is expected to enhance bacteriophages removal.^16,68,69^ This is valid for ACF_AR_; however, the ACF_OX+SOX_ have a greater amount of oxygen functional groups that might change their character to hydrophilic one. Carboxylic groups form intermolecular hydrogen bonds between groups ends when they are protonated, resulting in a decrease in hydrophilicity with pH drop.^70,71^ Lim *et al.* discussed the interactions between M13 bacteriophage and different functional groups, indicating that bacteriophages have fewer chances to create hydrogen bonds with carboxylic groups on a short timescale compared to hydroxyl groups at lower pH.^70,71^ This indicates that the contact time between the ACF material and the microorganism, as well as the type and concentration of oxygen surface functional groups, can significantly alter the physical interactions and is strongly affected by pH.

It can be assumed that MS2 bacteriophages were adsorbed on ACFs surfaces, rather than inactivated, because carbon itself is not recognized to possess antiviral characteristics and to interacts with virus capsid, *e.g.* carbon is known as a bioinert material applied in implants.^72^ This presented study did not reveal a strong MS2 adsorption interaction with ACFs, which resulted in facile desorption during further cartridge flushing. Reversible adsorption could be influenced by pH and presence of ions. Therefore, this aspect requires further consideration.^68,73^

### CuACF_OX+SOX_ and HCuACF_OX+SOX_

A high copper concentration was detected in the permeates, and this could be explained by the presence of a Tris – (tris(hydroxymethyl)aminomethane) buffer used for the preparation of the MS2 test solutions. Tris chelates metal ions, which in this study resulted in an enhanced release of copper from the ACFs Cu-composites, leading to the formation of ligand complexes: Cu(Tris)^2+^, Cu(Tris)_2_^2+^, Cu(Tris)_4_^2+^ or mixed ones,^74^ as well as destruction of the composites. The stable copper complexes formed with Tris did not interact with the MS2 bacteriophages remaining in the collected permeate. Therefore, further MS2 inactivation over time was not noted. Assuming that the structure of the tested composites was affected, only theoretical explanations of MS2 removal mechanisms can be discussed. Virus removal is due to a contribution of VdW forces, electrostatic adsorption, which occurred for HCuACF_OX+SOX_ at both pHs, as well as, an immediate interactions between copper and MS2 bacteriophages onto the composites surface^75–78^ that are influenced by copper species oxidation state, morphology (size, shape, distribution).^60,79^ As a comparison, Shimabuku *et al.* demonstrated that for 150 g of granular AC modified with 0.5 and 1 w/w% of Cu, a LRV equal to 0.33 and 0.39 LRV respectively.^23^ GAC modified with Al_2_O_3_ resulted in low efficiency of MS2 removal,^22^ while GAC modified with Ag and CuO exhibited 3 LRV for T4 bacteriophages^23^

This studies proves that fabricated Cu-composites are unstable, because of weak copper-ACFs bonding or removal of copper itself, being exceptionally delicate in water treatment applications, due to its dissolution, interaction or complexation by natural waters compounds.^80,81^ The copper virucidal properties are indisputable^34,35,82^ and highly beneficial. However, the copper modified ACFs presented in this study are suggested not to be considered in future studies of water treatment applications due to presence of weak copper–carbon bonds.

## Conclusions

ACFs were modified by a combination of HNO_3_ oxidation and Soxhlet extraction. The process did not markedly affect the structural and morphological features of ACFs and resulted in surface oxidation, functional groups formation, as well as an increase in microporosity. Synthesis of Cu-composites revealed the incorporation of copper onto the fibers' surface, as well as into the porous structure *via* copper ion exchange mechanism and coordination with surface functional groups followed by displacement of protons.

ACF_AR_ and ACF_OX+SOX_ exhibited a great potential towards MS2 bacteriophage removal reaching better virucidal performance (3.6 LRV) than AC in granular or powder forms. Moreover, ACF_OX+SOX_ fibers showed a 20% greater LRV than ACF_AR_, demonstrating the advantage of ACF surface functional groups incorporation. Further work on ACFs will include applications of higher amount of material, greater volumes of virus solution, bacteriophages that possess different isoelectric point (*e.g.* ΦX174 or fr)^83^ and more complex water compositions, *e.g.* natural organic matter, ions and humic acid, to understand the virial removal capacity and competitive adsorption processes occurring between the target virus and other waterborne species. Copper modified ACFs composites exhibited weak copper-carbon bonds and a high sensitivity to media composition, resulting in a release of metal. Therefore, at the present time, these materials are not suitable for implementation in water purification technologies.

## Author contributions

This manuscript was written through contributions of all authors. All authors have given approval to the manuscript final version.

## Conflicts of interest

There are no conflicts to declare.

## Supplementary Material

RA-011-D1RA06373A-s001
