# Supplementary material for: Virus removal from drinking water using modified activated carbon fibers
Source: RSC Adv. 2021 Sep 23;11(50):31547–56. doi: 10.1039/d1ra06373a (PMC9041606; doi:10.1039/d1ra06373a)
Supplement: RA-011-D1RA06373A-s001 [file RA-011-D1RA06373A-s001.pdf]

Electronic Supplementary Material (ESI) for RSC Advances.
This journal is © The Royal Society of Chemistry 2021

Supporting Information

Virus Removal from Drinking Water using Modified Activated Carbon Fibers

Kamila Domagala, [a,b*] *Jon Bell,*[a] *Nur Sena Yüzbasi,*[a] *Brian Sinnet,*[c] *Dariusz Kata,*[b] *Thomas Graule*[a]

[a] Laboratory for High Performance Ceramics, Empa, Swiss Federal Laboratories for Materials Science and Technology, Überlandstrasse 129 Dübendorf, Switzerland

[b] Faculty of Materials Science and Ceramics, AGH, University of Science and Technology, al. Adama Mickiewicza 30 Krakow, Poland

[c] Department of Process Engineering, Eawag, Swiss Federal Institute of Aquatic Science and Technology, Überlandstrasse 133 Dübendorf, Switzerland

KEYWORDS: activated carbon fibers, oxygen functional groups, porous structure, copper ion adsorption, virus removal, point of zero charge

Material characterization – details

Porous structure characterization: Approximately 100 mg of each sample was placed into the sample tube. The sample chamber was heated to 150 °C and evacuated to pressure of 13 μbar, which took 1 hour. Following this temperature and pressure stabilization phase, the samples were heated to 150 °C and outgassed for 2 hours. After degassing, samples were cooled to room temperature and weighed, in order to attain the dry weight. The sample tubes were attached to the instrument measuring ports and isothermal measurements were performed using a dewar filled with either: an ice-water mixture for CO_2 adsorption at 273 K or liquid nitrogen for N_2 adsorption at 77 K.

Pore Size Distribution Analysis: Pore size distribution analysis for both mesoporosity (2–50 nm) and microporosity (<2 nm) has been calculated from high-resolution N_2 sorption isotherms at 77K using the Barrett-Joyner and Halender model (BJH) (2–50 nm) and the Density Functional Theory (DFT) model (developed by J. Jagiello and J.P. Olivier), respectively (as shown in Figures S8 – S10). The BJH adsorption and desorption pore size distributions show that most of the surface area in the mesopore range exists between 2–10 nm. However, the mesoporosity does not contribute significantly to the total surface area, and is not significantly affected by the oxidation treatment. Additionally, DFT shows that these carbons are exclusively microporous with the majority of the surface area existing between approximately 1–3 nm, as shown in Figure S10. Analysis with the DFT model shows a trimodal distribution of pores, with the distributions centered at pore widths equal to 1.6 nm, 1.9 nm and 2.3 nm for the as-received carbon ACF_{AR}. Soxhlet extraction of as-received carbon (ACF_{AR+SOX}) does not affect the distribution significantly. However, oxidation (ACF_{OX}) and oxidation followed by Soxhlet extraction (ACF_{OX+SOX}) affects the DFT pore size distribution significantly, with a loss of the trimodal

character and a vast reduction in micropore surface area (as shown in Figure S10). This indicates that functionalization with HNO_3 introduces surface oxygen functional groups that significantly block and reduce the micropores in activated carbon fibers.

Elemental Analysis: For the analysis of CHNS elements, the system was calibrated with the standard- Methionine, while ACF samples (approximately 0.4 mg) were weighed in tin crucibles and then loaded into the Flash Smart analyzer. For the analysis of the oxygen content, reference samples of the BBOT standard were used for equipment calibration. Samples (approximately 1 mg) were weighed in silver crucibles and loaded into analyzer.

Point of zero charge: The pH of a series of 0.01 M NaCl solutions was adjusted from 1 - 12 by adding either HCl or NaOH. Solutions were degassed by bubbling N_2 gas at 298 K to remove dissolved CO_2 until the initial pH stabilized. 75 mg of ACFs were added to 25 mL of the solution. The final pH was recorded after 24 hours. The point at which initial pH and final pH values were equal was taken as the point of zero charge.

Boehm titration: 250 mg of ACFs into 25 mL of three reaction bases of 0.05 M NaOH, 0.05 M $NaHCO_3$ and 0.05 M Na_2CO_3[1–3]. The samples were stirred for 24 h in order to reach acid-base reaction equilibrium. Subsequently, the suspensions were filtered (PVDF membrane 0.1 μm, 47 mm, Hawach Scientific Co., Ltd) and 10 mL aliquots of filtrate were collected. The aliquots were acidified by addition of 20 mL of standardized 0.05 M HCl. 20 mL of 0.05 M HCl for aliquots of the NaOH, $NaHCO_3$ reaction base and 30 mL of 0.05 M HCl for Na_2CO_3. The acidified sample was degassed via bubbling N_2 for 2 h to expel dissolved CO_2 and then back-titrated with a standardized solution of 0.05 M NaOH, while being continually saturated with N_2. The endpoints were determined using a pH Meter (FiveEasyPlus, Mettler Toledo) and phenolphthalein as an indicator. All steps were performed at room temperature.

X-ray photoelectron spectroscopy: The measurements were performed using Al Kα (1254 eV) radiation and an analyzer pass energy of 100 eV. The spectra ware recorded in normal emission geometry with an energy resolution of 0.9 eV and the ultra-high vacuum (UHV) conditions of 10^{-9} mbar. The area of analysis was approximately 3 mm^2 while depth of analysis was about 10 nm. The spectra were analyzed with the use of CasaXPS 2.3.15 software. The calculations of elements at the sample surface were performed with QUASES-IMFP-TPP2M Ver 2.2 software according to Tanuma et al.[4] Deconvolution of the spectrum on spectrum components was done according to the work by J.A. Leiro et al.[5]. Sample (the bunch of fibers) was mounted and positioned at the dedicated holder and pumped out to high vacuum then transferred into UHV chamber.

Raman Spectroscopy: Spectra were recorder in range 120-3500 cm^{-1}. ACFs substrates were placed on glass disc and directly measured. OriginPro 2018 was used for peak fitting in the range of 800–1800 cm^{-1} (first-order region). Background subtractions were performed by forming a baseline using the regions of 800–950 cm^{-1} and 1750–1800 cm^{-1}.

Scanning electron microscope: ACFs were directly placed on the carbon double-side adhesive tape and analyzed. Composites were sputtered with a layer of a conductive carbon

X-ray diffraction: The scans were acquired in the 2θ range of 5−80° with a step size of 0.016° and a scanning speed of $0.021°s^{-1}$. Phase compositions of the spectra were analyzed with HighScore Plus software.

ICP-MS of ACFs used for synthesis and composites: ICP-MS (ICP-MS 7500CE, Agilent) was applied to determine copper concentrations in as-received fibers (ACF_{AR}), fibers used for composites synthesis (ACF_{OX+SOX}), as well as in manufactured composites $CuACF_{OX+SOX}$ and HCu ACF_{OX+SOX}. ACFs and respective composites were acid digested using a microwave digestion system ultraCLAVE (MLS GmbH) prior to analysis. Digestions were performed in 40 mL HNO_3 (65 %) and 5 mL H_2O_2, (30 %), for 50 mg samples. The ultra-CLAVE ran using the

following steps: Step 1: 25 °C to 160 °C in 10 min.; Step 2: 160 °C to 240 °C in 9 min.; Step 3: 240 °C for 10 min. After cooling down, the solution was removed and filled up to 50 mL with nanopure water.

Cartridge - details

The glass cartridge was specially made for flow test, having following dimensions: d_{in}=8 mm, d_{out}=14.4 mm, l=35 mm. In the polymer inlet and outer caps of the cartridge, cut glass fiber filters (0.4 µm, Macherey-Nagel) were placed to avoid fibers being released from the system.

Tables

Tab. S1 Composition of solutions used with DAL method with MS2 bacteriophages.

**Soft Agar (0.7%)**		
Nanopure H_2O, mL	Tryptic Soy Agar, g	$MgSO_4$, g
1000	32	0.60

**Hard Agar (1.5%)**		
Nanopure H_2O, mL	Tryptic Soy Agar, g	$MgSO_4$, g
1000	40	0.60

**Broth**						
Nanopure H_2O, mL	Tryptone, g	Yeast extract, g	NaCl, g	Glucose, g	$CaCl_2$, g	$MgSO_4$, g
1000	1	0.10	0.80	0.10	0.03	0.015

**Virus Dilution Buffer (VDB)**		
Nanopure H_2O, mL	$NH_2C(CH_2OH)_3$, g	$MgSO_4$, g
1000	2.50	0.60

Tab. S2 Pore structure and BET characterization by CO_2 and N_2 adsorption-desorption of activated carbon fibers.

**Carbon sample**	**Pore volume, cm^3g^{-1}**		**V_{CO2}/V_{N2}**	**x_0^c, nm**	**x_0^d, nm**	**SSA, m^2g^{-1}**
	V_{CO2}^a	V_{N2}^b				
ACF_{AR}	0.108	0.453	0.238	1.08	0.26	1677
ACF_{AR+SOX}	0.130	0.468	0.279	1.06	0.29	1769
ACF_{OX}	0.137	0.186	0.740	1.06	0.79	698
ACF_{OX+SOX}	0.141	0.397	0.356	0.96	0.34	1652

a obtained from an intercept of D-R plot
b obtained from Langmuir model at $p/p^0=1$
c mean radius of the micropore (N_2 at 77 K)
d mean radius of the micropore (CO_2 at 273 K)

Tab. S3 Functional groups concentrations determined by Boehm titration of activated carbon fiber.

**Carbon sample**	**Phenolic, mmol/g**	**Lactone, mmol/g**	**Carboxylic, mmol/g**	**Total amount, mmol/g**
ACF_{AR}	0.36	0.00	0.10	0.46
ACF_{AR+SOX}	0.11	0.31	0.30	0.72
ACF_{OX}	0.04	1.01	2.26	3.31
ACF_{OX+SOX}	0.83	0.61	2.04	3.48

Tab. S4: Adsorption parameters obtained from Langmuir equation for $Cu^{2+}{}_{(aq.)}$ ion adsorption;

**Carbon sample**	**pH_{init}**	**Langmuir equation**		
		**n_m, mmol·g^{-1}**	**K, L·mmol^{-1}**	**R^2**
ACF_{AR}	unchanged(4.3)	2.00	0.036	0.941
	4.0	0.99	0.118	0.975
	1.0	0.93	0.095	0.934
ACF_{OX+SOX}	unchanged (4.3)	1.24	0.451	0.993
	4.0	1.75	0.269	0.996
	2.0	0.90	0.551	0.921
	1.0	0.88	0.251	0.919

Tab. S5 Pore volumes and specific surface areas of activated carbon fibers based composites.

**ACFs composite**	**Pore volume, cm^{3}g^{-1}**		**V_{CO2}/V_{N2}**	**SSA, m^{2}g^{-1}**
	V_{CO2}[a]	V_{N2}[b]		
$CuACF_{OX+SOX}$	0.067	0.160	0.420	584
$HCuACF_{OX+SOX}$	0.077	0.181	0.425	635

a obtained from an intercept of DR plot
b obtained from Langmuir model at $p/p^0=1$

Tab. S6 XPS profiles of functional groups for copper impregnated ACFs before and after heat-treatment.

**ACFs composite**	**Components from C 1s profile (at.%)**					
	**A: C-C**	**B: C-O + C-N**	**C: C=O + C=N**	**D: COOH**	**E: CO_3**	**X: C-Me + NC-Me**
$CuACF_{OX+SOX}$	54.50	5.69	4.11	9.22	3.89	2.33
$HCuACF_{OX+SOX}$	59.00	7.77	4.93	6.65	4.42	4.04

**ACFs composite**	**Components from O 1s profile (at.%)**			
	**A: O-Me**	**B: OH**	**C: O-C + H_2O**	**D: O-C=**
$CuACF_{OX+SOX}$	0.63	5.85	10.65	0.90
$HCuACF_{OX+SOX}$	0.47	3.87	5.34	0.77

**ACFs composite**	**Components from N 1s profile (at.%)**				
	**A: N-Me + Me-CN**	**B: N-C**	**C: NH_4^+**	**D: N^{3+}-O; NO_2^-**	**E: N^{5+}-O-C; NO^{3-}**
$CuACF_{OX+SOX}$	0.35	0.63	0.34	0.26	0.06
$HCuACF_{OX+SOX}$	0.18	0.73	0.14	0.28	0.05

**ACFs composite**	**Components from Cu 2p profile (at.%)**			
	**A: Cu^+-CN**	**B: Cu^{2+}-O, CuO**	**C: Cu^{2+}-OH, Cu^{2+} in salts**	**X: Cu-C**
$CuACF_{OX+SOX}$	0.41	0.18	0.05	0.00
$HCuACF_{OX+SOX}$	1.04	0.17	0.09	0.08

Figures

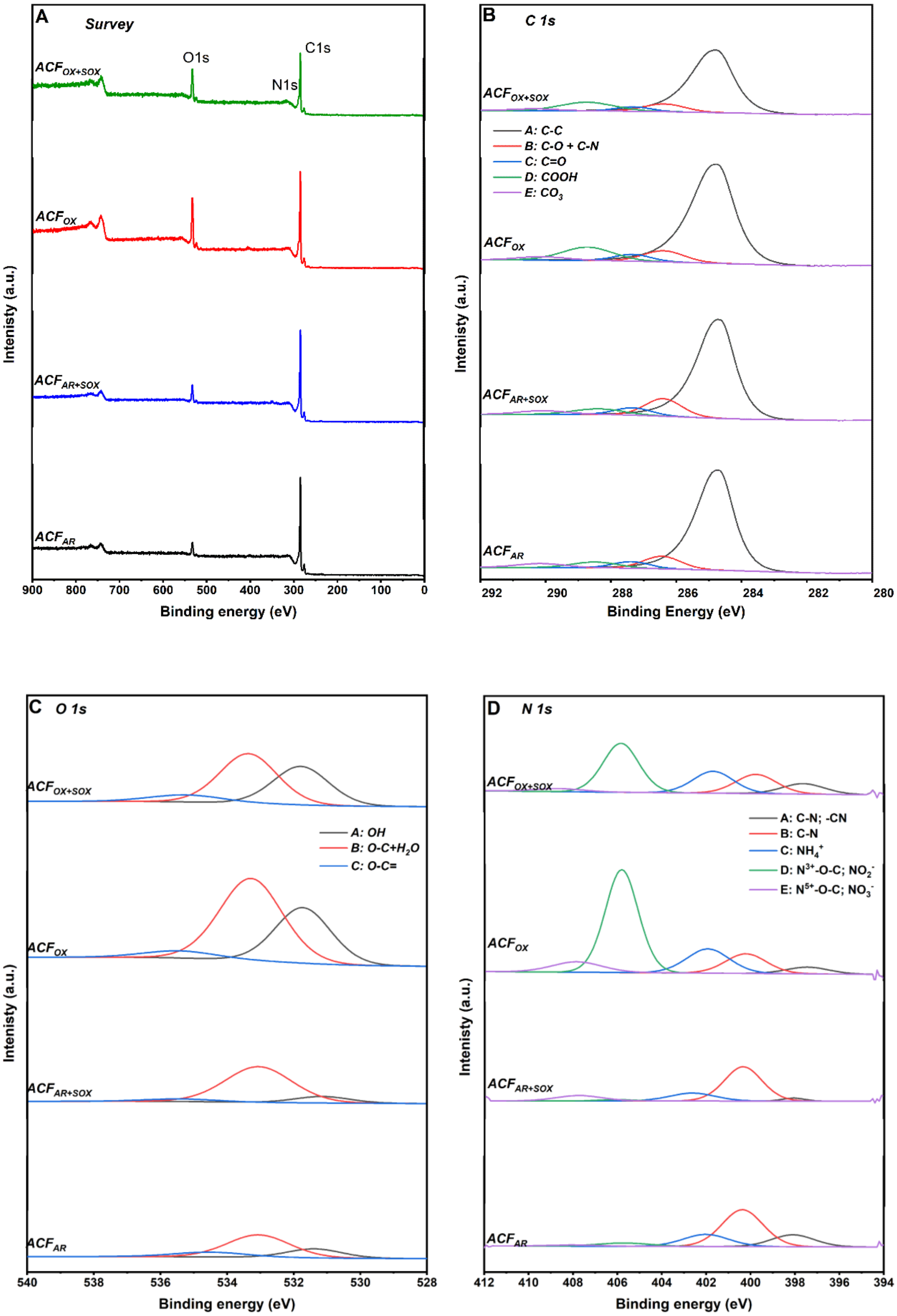

Fig. S1 XPS spectrum a) Survey; b) C 1s; c) O 1s; d) N 1s of activated carbon fibers.

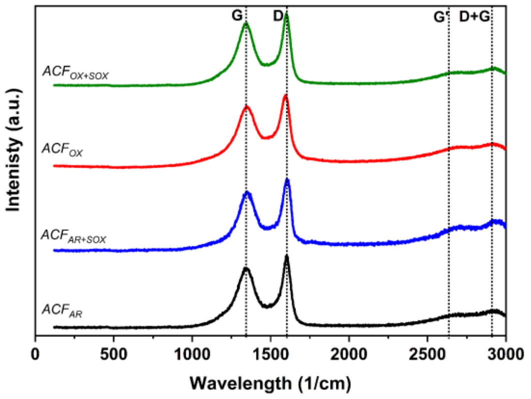

Fig. S2 Raman spectroscopy of all activated carbon fibers.

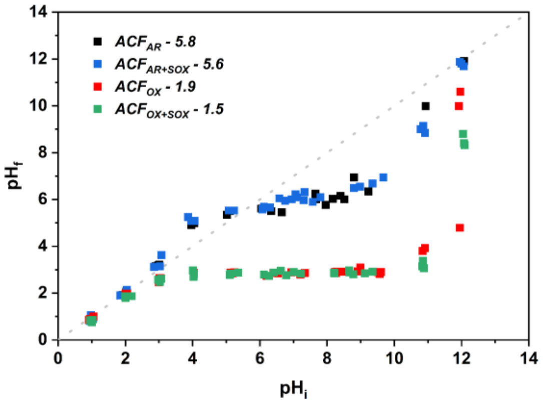

Fig. S3 Graph of final pH versus initial pH of activated carbon fibers.

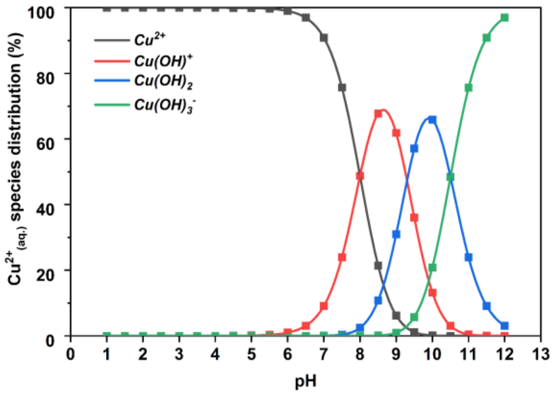

Fig. S4 Speciation diagram of copper.

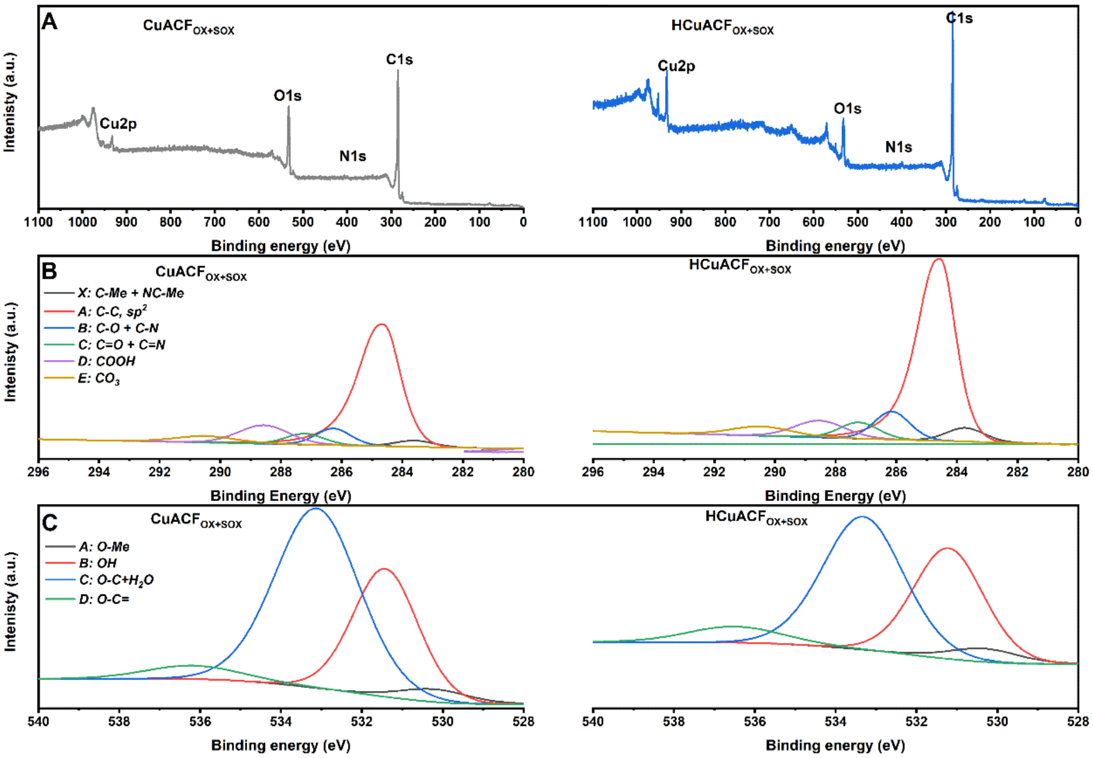

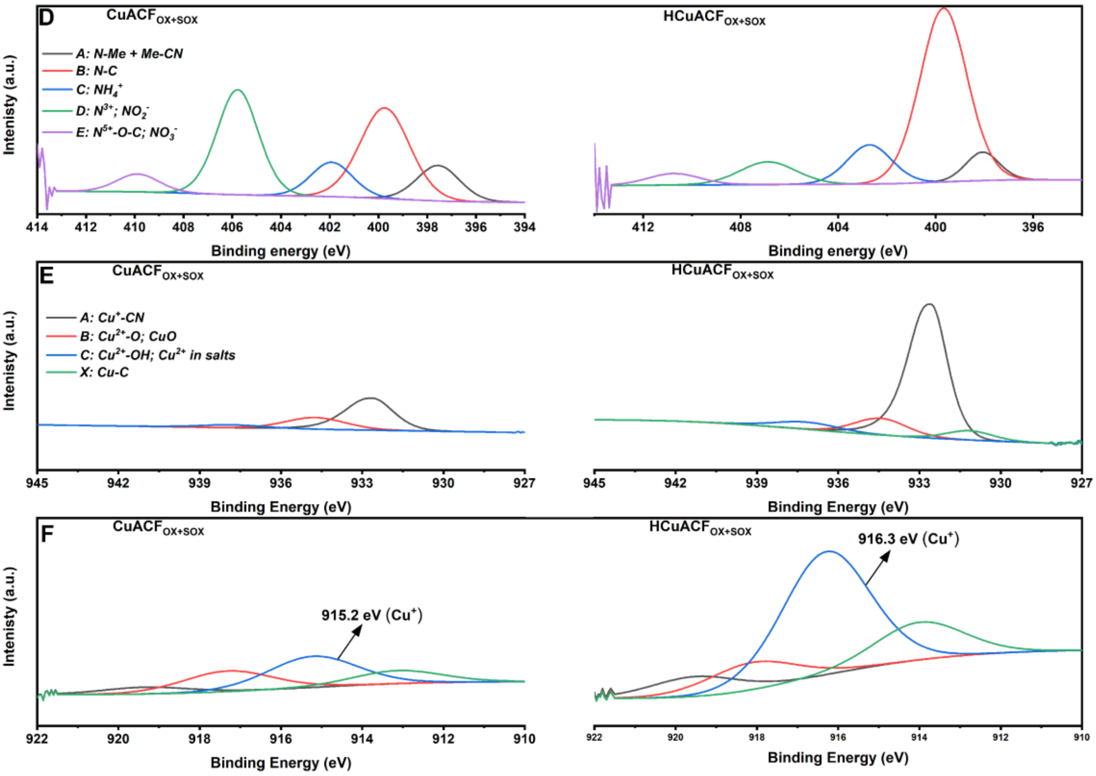

Fig. S5 XPS spectra a) Survey; b)C 1s; c) O 1s; d) N 1s; e) Cu 2p; f) Cu LVV XANES of ACF_{OX+SOX} based composites.

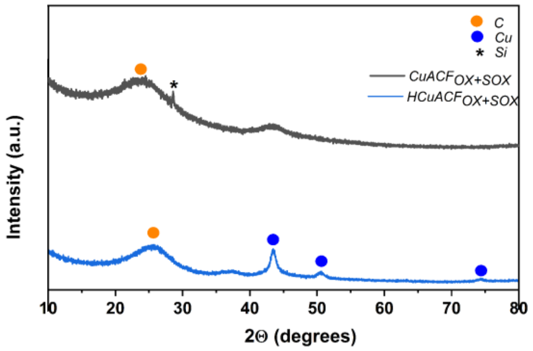

Fig. S6 XRD patterns of activated carbon fibers based composites (Si peak comes from the sample holder.

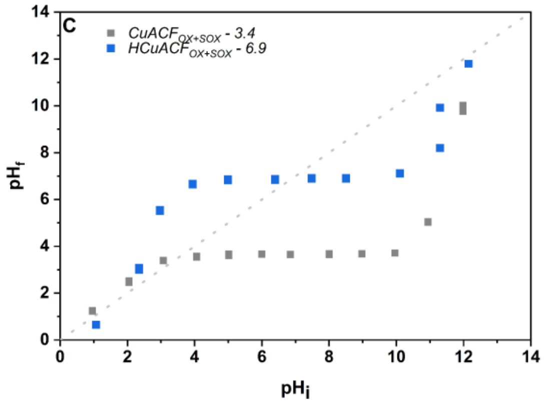

Fig. S7 Graph of final pH versus initial pH of composites.

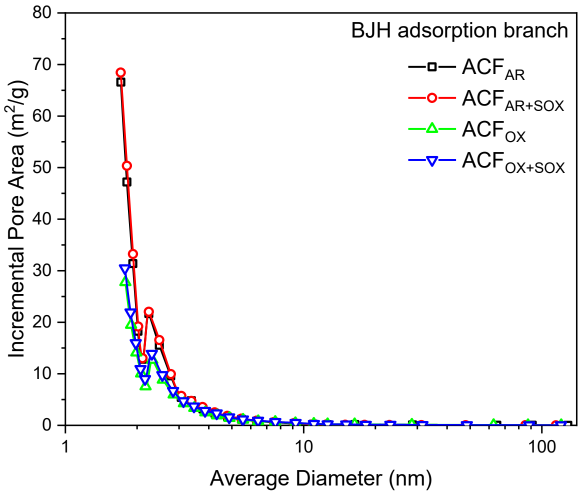

Fig. S8 Pore size distribution calculated from adsorption branch using the BJH model.

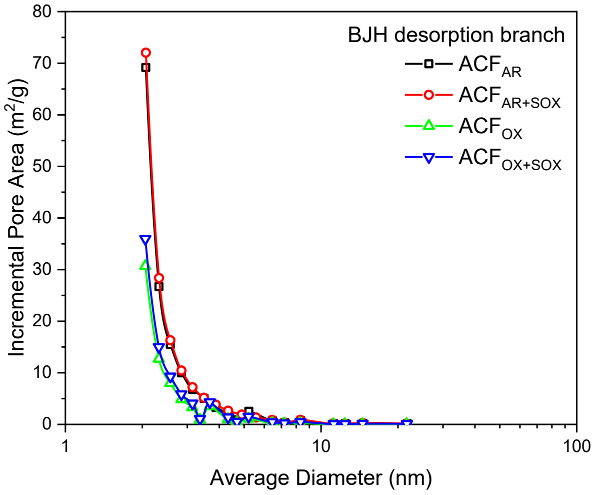

Fig. S9 Pore size distribution calculated from desorption branch using the BJH model.

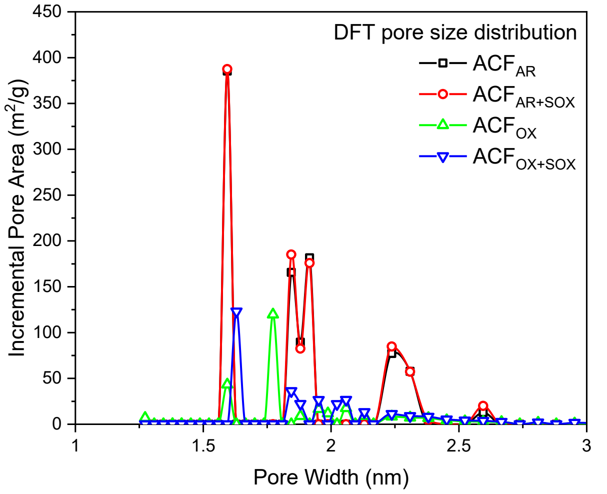

Fig. S10 Pore size distribution calculated from the density functional theory (DFT) model.

References

1 A. M. Oickle, S. L. Goertzen, K. R. Hopper, Y. O. Abdalla and H. A. Andreas, *Carbon N. Y.*, , DOI:10.1016/j.carbon.2010.05.004.

2 S. L. Goertzen, K. D. Thériault, A. M. Oickle, A. C. Tarasuk and H. A. Andreas, *Carbon N. Y.*, 2010, **48**, 1252–1261.

3 M. Voll and H. P. Boehm, *Carbon N. Y.*, 1971, **9**, 481–488.

4 S. Tanuma, C. J. Powell and D. R. Penn, *Surf. Interface Anal.*, 1994, **21**, 165–176.

5 J. A. Leiro, M. H. Heinonen, T. Laiho and J. G. Baitrev, *J. Electron Spectrosc. Relat. Phenom.*, 2003, **128**, 205–213.
